# Epigenetic silencing of *TGFBI* confers resistance to trastuzumab in human breast cancer

**DOI:** 10.1186/s13058-019-1160-x

**Published:** 2019-07-05

**Authors:** Sònia Palomeras, Ángel Diaz-Lagares, Gemma Viñas, Fernando Setien, Humberto J. Ferreira, Glòria Oliveras, Ana B. Crujeiras, Alejandro Hernández, David H. Lum, Alana L. Welm, Manel Esteller, Teresa Puig

**Affiliations:** 1 New Therapeutics Targets Lab (TargetsLab), Department of Medical Sciences, University of Girona, E-17071 Girona, Catalonia Spain; 2 Cancer Epigenetics and Biology Program (PEBC), Bellvitge Biomedical Research Institute (IDIBELL), Hospitalet de Llobregat, Barcelona, Catalonia Spain; 3 Cancer Epigenomics, Translational Medical Oncology (Oncomet), Health Research Institute of Santiago (IDIS), University Clinical Hospital of Santiago(CHUS/SERGAS), CIBERONC, Santiago de Compostela, Spain; 4 Medical Oncology Department, Catalan Institute of Oncology (ICO), Girona, Catalonia Spain; 5 Girona Biomedical Research Institute (IDIBGI), E-17071 Girona, Catalonia Spain; 6 Pathology Department, Dr. Josep Trueta Hospital and Catalan Institute of Health (ICS), E-17071 Girona, Catalonia Spain; 7 Laboratory of Epigenomics in Endocrinology and Nutrition, Health Research Institute of Santiago (IDIS), University Clinical Hospital of Santiago (CHUS/SERGAS), Santiago de Compostela, Spain; 8 CIBER Fisiopatologia de la Obesidad y Nutricion (CIBERobn), Santiago de Compostela, Spain; 9 Department of Oncological Sciences, Huntsman Cancer Institute, University of Utah, Salt Lake City, USA; 10 Centro de Investigacion Biomedica en Red Cancer (CIBERONC), Madrid, Spain; 11 Physiological Sciences Department, School of Medicine and Health Sciences, University of Barcelona (UB), Barcelona, Catalonia Spain; 12 Institucio Catalana de Recerca i Estudis Avançats (ICREA), Barcelona, Catalonia Spain; 13 Josep Carreras Leukaemia Research Institute (IJC), Badalona, Barcelona, Catalonia Spain

**Keywords:** HER2+ breast cancer, Trastuzumab resistance, DNA methylation, TGFBI

## Abstract

**Background:**

Acquired resistance to trastuzumab is a major clinical problem in the treatment of HER2-positive (HER2+) breast cancer patients. The selection of trastuzumab-resistant patients is a great challenge of precision oncology. The aim of this study was to identify novel epigenetic biomarkers associated to trastuzumab resistance in HER2+ BC patients.

**Methods:**

We performed a genome-wide DNA methylation (450K array) and a transcriptomic analysis (RNA-Seq) comparing trastuzumab-sensitive (SK) and trastuzumab-resistant (SKTR) HER2+ human breast cancer cell models. The methylation and expression levels of candidate genes were validated by bisulfite pyrosequencing and qRT-PCR, respectively. Functional assays were conducted in the SK and SKTR models by gene silencing and overexpression. Methylation analysis in 24 HER2+ human BC samples with complete response or non-response to trastuzumab-based treatment was conducted by bisulfite pyrosequencing.

**Results:**

Epigenomic and transcriptomic analysis revealed the consistent hypermethylation and downregulation of *TGFBI*, *CXCL2*, and *SLC38A1* genes in association with trastuzumab resistance. The DNA methylation and expression levels of these genes were validated in both sensitive and resistant models analyzed. Of the genes, *TGFBI* presented the highest hypermethylation-associated silencing both at the transcriptional and protein level. Ectopic expression of TGFBI in the SKTR model suggest an increased sensitivity to trastuzumab treatment. In primary tumors, *TGFBI* hypermethylation was significantly associated with trastuzumab resistance in HER2+ breast cancer patients.

**Conclusions:**

Our results suggest for the first time an association between the epigenetic silencing of *TGFBI* by DNA methylation and trastuzumab resistance in HER2+ cell models. These results provide the basis for further clinical studies to validate the hypermethylation of *TGFBI* promoter as a biomarker of trastuzumab resistance in HER2+ breast cancer patients.

**Electronic supplementary material:**

The online version of this article (10.1186/s13058-019-1160-x) contains supplementary material, which is available to authorized users.

## Background

Breast cancer (BC) is the most common cancer in women worldwide and the leading cause of cancer deaths for women [1]. Approximately 15–20% of patients with this tumor overexpress the human epidermal growth factor receptor 2 (HER2) protein [2]. HER2 can activate downstream signaling cascades that induce cell proliferation through the Ras-mitogen-activated protein kinase (MAPK) pathway and inhibit cell death through the phosphatidylinositol 3′-kinase (PI3K)/protein kinase B (Akt)/mammalian target of the rapamycin (mTOR) pathway [3, 4]. Characterizing HER2 as a proto-oncogene, a poor prognostic marker, and eventually as a therapeutic target has dramatically changed BC classification, risk assessment, and treatment [5]. The development of powerful targeted therapies directed specifically at HER2 has improved the survival of patients with both early-stage and metastatic BC.

Trastuzumab (Herceptin®; Genentech, Inc., South San Francisco, CA, USA) is a humanized monoclonal antibody that selectively binds with high affinity to the extracellular domain of human HER2 protein and was the first targeted drug approved for treating HER2+ BC [6]. Although there are currently other anti-HER2 agents available, trastuzumab remains the gold standard for treating this subtype of BC. Despite the clinical benefits trastuzumab treatment in HER2+ BC brings, a large percentage of patients display primary or acquired resistance to the drug. In the last few years, several studies have focused on identifying the molecular mechanisms of trastuzumab resistance, such as aberrant activation of downstream signaling pathways [7] or the HER2 carboxyl-terminal fragments (CTF), also known as p95HER2, which are frequently found in HER2-expressing BC cell lines and tumors [8]. Despite these efforts, the complete picture of the molecular mechanisms triggering trastuzumab resistance in BC remains unclear. Therefore, clarifying these mechanisms and identifying new resistance biomarkers is essential in the advance towards precision oncology in BC and the quest for new treatment options for those patients who do not respond to trastuzumab therapy.

DNA methylation is the most well-known epigenetic modification in humans and has been implicated in regulating the expression of a great variety of critical genes in cancer [9]. For this reason, DNA methylation status has emerged as one of the most promising epigenetic biomarkers for several types of cancer, including BC [10]. These epigenetic markers can be useful in detecting tumors earlier or identifying patients with an increased risk of cancer, as well as evaluating disease progression or predicting the response to anticancer drugs [11]. In the last few years, some significant genes that are inactivated by promoter methylation in BC have been identified, including BRCA1 [12] and RASSF1A [13]. However, the analysis of hypermethylated genes in association with trastuzumab resistance is still a largely unexplored field that holds great potential.

The aim of this study was to evaluate the implication DNA methylation has in trastuzumab resistance and to identify epigenetically regulated genes with potential clinical value as biomarkers for trastuzumab resistance in HER2+ BC patients. To this purpose, we employed an integrative approach with genome-wide DNA methylation (450K array) and a transcriptomic analysis (RNA-Seq) in trastuzumab-sensitive and trastuzumab-resistant cell line models (SK and SKTR). In vitro results were validated in a cohort of 24 HER2+ tumor samples from patients experiencing both sensitivity and resistance to trastuzumab-based neoadjuvant therapy settings. Here, we determine epigenetic inactivation of the *TGFBI* gene by promoter CpG island hypermethylation, which are CpG-rich regions of DNA that are often associated with the transcription start sites of genes, with possible implications for trastuzumab-resistant BC pathways [14]. The hypermethylation of *TGFBI* also suggest its potential clinical usefulness as a biomarker for trastuzumab resistance in HER2+ BC patients.

## Methods

### Cell culture

SKBr3 (SK) and AU565 (AU) HER2+ breast carcinoma cells were obtained from Eucellbank (University of Barcelona, Spain) [15] and the American Type Culture Collection (ATCC, Rockville, MD, USA). SKBr3 and AU565 cells were routinely grown in McCoy’s (Gibco) and Dulbecco’s modified Eagle’s medium (DMEM; Gibco), respectively, and supplemented with 10% FBS (HyClone Laboratories), 1% l-glutamine (Gibco), 1% sodium pyruvate (Gibco), and 100 U/mL penicillin/streptomycin (HyClone Laboratories). Cell lines were kept at 37 °C and 5% CO_2_ atmosphere. Long-term trastuzumab-resistant SK cells (SKTR) and AU565 cells (AUTR) had been previously developed in our laboratory [16, 17]. Resistance was confirmed with cell viability assays. The trastuzumab-resistant SKTR and AUTR cells were maintained in 2 μM of trastuzumab, i.e., a concentration in which parental cells were not viable.

### Patients and tissue samples

*TGFBI* promoter methylation levels were retrospectively evaluated in tumor samples from 24 patients diagnosed with HER2+ BC at the Dr. Josep Trueta University Hospital, Girona (Spain) between 2007 and 2015. The patients were selected from the hospital’s pharmacy database. The selection criterion included patients with early or locally advanced HER2+ BC who had received neoadjuvant treatment with trastuzumab and chemotherapy. Twenty patients had no response or partial response and 4 patients had complete response to trastuzumab *plus* chemotherapy. For all patients, hematoxylin and eosin (H&E)-stained slides from formalin-fixed paraffin-embedded (FFPE) tumor blocks were examined to determine the representative areas of the invasive tumor. Estrogen receptor (ER), progesterone receptor (PR), and HER2 expression had been previously analyzed in the tumors using immunohistochemistry (IHC). For each patient, clinical and histopathological features were obtained: age, stage (TNM classification [18]), histological grade (Bloom-Richardson grading system), menopause status, type of surgery, and relapse.

### 5-Aza-2′-deoxycytidine treatment

Epigenetic signatures are characterized by a very dynamic nature, where DNA methylation has often been shown as a reversible mechanism of transcriptional control by inhibition of enzymes such as the DNA methyltransferases [19]. We performed reactivation treatments using the demethylating agent, 5-aza-2′-deoxycytidine (5-aza-dC, Sigma-Aldrich, St Louis, MO) at 3 μM and 5 μM of 5-aza-dC for 72 h. The medium was changed every day to promote DNA demethylation.

### DNA and RNA isolation procedures

Genomic DNA extraction from cell lines or formalin-fixed paraffin-embedded (FFPE) core biopsies (10 μm) and tissue sections (5 μm) using a QIAamp DNA Mini Kit and Deparaffinization Solution with a QIAamp DNA FFPE Tissue Kit (Qiagen, Hilden, Germany), respectively, were carried out following the manufacturer’s instructions. For the RT-PCR experiments, cells were washed with PBS and then suspended in 1 mL of Qiazol (Qiagen Hilden, Germany) was added. Total RNA was isolated using a GeneJET RNA Purification Kit (Thermo Fisher Scientific) following the instructions provided by the manufacturer. All DNA and RNA samples were quantified using a NanoDrop 2000 Spectrophotometer (Thermo Fisher Scientific).

DNA was bisulfite-modified using an EZ DNA Methylation-Gold Kit (Zymo Research) in accordance with the manufacturer’s recommendations.

### DNA methylation array

Genome-wide DNA methylation analysis was performed using an Illumina 450K DNA methylation microarray (Infinium HumanMethylation450 BeadChip) as previously described [20]. This technique is an epigenomic approach that allows analyzing the methylation profile of the human genome. Bisulfite-converted DNA from SK and SKTR models were used to hybridized on an Illumina Infinium HumanMethylation450 (450K) BeadChip array, following the Illumina Infinium HD Methylation protocol. Sex chromosomes were excluded from the analysis because they usually represent a high source of variation in DNA methylation levels [21]. Data was deposited into the NCBI Gene Omnibus, accession number: GSE123754.

### RNA sequencing

DNA methylation is an epigenetic mechanism that usually affects CpG islands and promoters regulating the expression of the genes. For this reason, a transcriptomic analysis by RNA-Seq was performed. Library construction was performed using an Illumina TruSeq Stranded mRNA Sample Preparation Kit (CAT. No. RS-122-2101, RS-122-2102). Following the transfer of the flow cell to an Illumina HiSeq instrument, a 101-cycle paired-end sequence run was performed using the HiSeq SBS Kit v4 sequencing reagents (FC-401-4002). A detailed description of the analysis is provided in the Additional file 1. Sequencing data have been posted in the Gene Expression Omnibus (GEO) database under accession number GSE114575.

### Bisulfite pyrosequencing

To validate the results obtained from the arrays, bisulfite pyrosequencing analyses were performed. The primers for the PCR amplification and sequencing were designed with the PyroMark assay design software version 2.0.01.15. DNA-bs was amplified by PCR under standard conditions with biotinylated primers (see Additional file 1: Table S1) to convert the PCR product into single-stranded DNA templates. We used a Vacuum Prep Tool (Biotage, Sweden) to prepare single-stranded PCR products following the manufacturer’s instructions. The PCR products were observed in 2% agarose gels before pyrosequencing. Reactions were performed in a PyroMark Q96 System version 2.0.6 (Qiagen, Hilden, Germany) using appropriate reagents and protocols. The methylation value was obtained from the average of the CpG dinucleotides included in the sequences analyzed using a Pyro Q-CpG 1.0.9 (Qiagen, Hilden, Germany).

### Methylation-specific PCR (MSP)

DNA methylation was also validated using the methylation-specific PCR (MSP) using a set of primers designed by the Methyl Primer Express program for each gene analyzed. For each sample, two PCR reactions were produced using the same DNA template: one reaction with methylated primers (M: methylated) and the other with unmethylated primers (U: unmethylated). Commercial methylated human male genomic DNA (CpGenome Universal Methylated DNA, Millipore) was used as a methylated positive control (IVD), and DNA from normal lymphocytes (NL) as a positive control for unmethylated alleles. Primer sequences can be found in Additional file 1: Table S2.

### Gene expression analysis (qRT-PCR)

To confirm the differential regulation of *TGFBI*, *CXCL2* and *SLC38A1* observed in SK and SKTR models used gold standard methodologies for gene expression such as quantitative real-time PCR. Total RNA was reverse-transcribed into cDNA using a High Capacity cDNA Archive Kit (Applied Biosystems). Gene expression levels of selected genes were assessed using a LightCycler 480 Real-time PCR System (Roche) with a LightCycler 480 SYBR Green I Master (Roche). Primers are depicted in Additional file 1: Table S3*.* RT-PCR analyses were performed at least four times, and each gene was run in triplicate. GAPDH was used as an endogenous control to enable normalization.

### Short hairpin interference and ectopic expression assays

For the TGFBI long-term knockdown, two different ShRNAs were specifically designed against *TGFBI* mRNA (NM_000358) in two different loci, by considering a 19-base target sequence and inserted in the pLVX-shRNA2 vector (Clontech). For stable overexpression, the cDNA sequence of TGFBI was amplified from SKWT cDNA and cloned into the pLVX-IRES-tdTomato vector (Clontech). This construction (wild type) was then used as a template to create its mutated version following a PCR-based strategy. Mutations were performed in the RGD domain and the NKDIL (amino acids 354–358), YH18 (amino acids 563–580), and EPDIM (amino acids 617–621) motif in the second and fourth FAS-1 domains [22]. Lentiviral production was performed using the protocol described in Additional file 1. After lentiviral transduction, ZsGreen1-positive or tdTomato-positive cells were sorted by flow cytometry.

### Cell viability assays

Cell viability was determined using a 3-(4, 5-dimethyl-2-thiazolyl)-2, 5-diphenyl-2 h-tetrazolium bromide (MTT) assay. Briefly, cells were plated in their growth medium in 96-well plates at a cell density of 1.5 × 10^3^ cells per well. After 24 h, the growth medium was removed, and, 100 μL of fresh medium containing the corresponding concentration of trastuzumab was added to each well for 5 days. Cell viability was measured using the standard colorimetric MTT assay as previously described [16]. Using the multi-well plate reader Benchmark Plus (Bio-Rad), absorbance was determined to be 570 nm. Data presented are from three separate wells per assay, and the assay was performed at least three times.

### Western blot analysis

We previously demonstrated that different activation patterns of some HER receptors and their downstream signaling proteins are associated with the molecular mechanisms of acquired trastuzumab resistance (SK and SKTR) [16]. For this reason, a panel of the different proteins were analyzed in our models using Western blot analysis. The parental (SK and AU) and resistant (SKTR and AUTR) models were synchronized by starvation in serum-deprived medium (0.5% FBS) for 24 h. Cells were lysed in ice-cold lysis buffer (Cell Signaling Technology, Inc.) with 100 μg/mL phenylmethylsulfonylfluoride (PMSF). Protein concentration was determined with Lowry (DC Protein Assay, Bio-Rad). Equal amounts of protein were heated in LDS Sample Buffer with Sample Reducing Agent (Invitrogen) for 10 min at 70 °C, separated on SDS-PAGE and transferred to nitrocellulose membranes. Protein was detected using primary antibodies (Additional file 1: Table S5). Secondary antibodies, α-tubulin and β-actin, conjugated to horseradish peroxidase were used. The immune complexes were detected using a chemiluminescent HRP substrate [SuperSignal™ West Femto Maximum Sensitivity Substrate (Thermo Scientific™ Inc.) or Immobilon Western HRP Substrate (Millipore Sigma)] and in a Bio-Rad ChemiDoc™ MP Imaging System. Western blot analyses were repeated at least three times and representative results are shown.

### Statistical analysis

Data were analyzed with Student’s *t* test when two groups were being compared or with one-way analysis of variance (ANOVA) followed by Tukey’s honest significant difference (HSD) post hoc test or Tamhane’s T2 post hoc test for multiple comparisons. The non-parametric Kruskal-Wallis test was used when data did not follow normal distribution. When two groups were being compared, the data were analyzed with the Mann-Whitney *U* tests for non-normally independent variables; otherwise, the Kruskal-Wallis test was used for more than two groups. In the patient cohort, we analyzed the *TGFBI *promoter methylation status and its association with the clinical-histopathological characteristics. Patient data were summarized as median (first quartile-third quartile) for continuous variables, and frequencies and percentages for categorical variables. The potential association between clinical-histopathological characteristics and levels of *TGFBI* methylation (low ≤ 20%, high ≥ 20%) or differences in *TGFBI *methylation before and after treatment were analyzed using the Mann-Whitney *U* and Kruskal-Wallis tests for continuous variables and the chi-square or Fisher exact tests, as appropriate, for categorical variables. The correlation between variables was observed using Spearman’s rho coefficient. Characteristic (ROC) curves were used to assess the predictive capacity of the TGFBI marker. ROC curve analysis was performed using MedCalc software Version 18.11.3. The Hosmer-Lemeshow test and calibration plot was assessed with MedCalc and STATA software. Levels of significance were set at *p* < 0.05 and are represented by asterisks, as follows: *p* < 0.05 (denoted as *), *p* < 0.01 (denoted as **), and *p* < 0.001 (denoted as ***). The statistical analysis was performed using the IBM SPSS software (Version 21.0; SPSS Inc.)

## Results

### Genome-wide DNA methylation analysis in trastuzumab-sensitive (SK) and trastuzumab-resistant (SKTR) breast cancer models

**Fig. 1:**
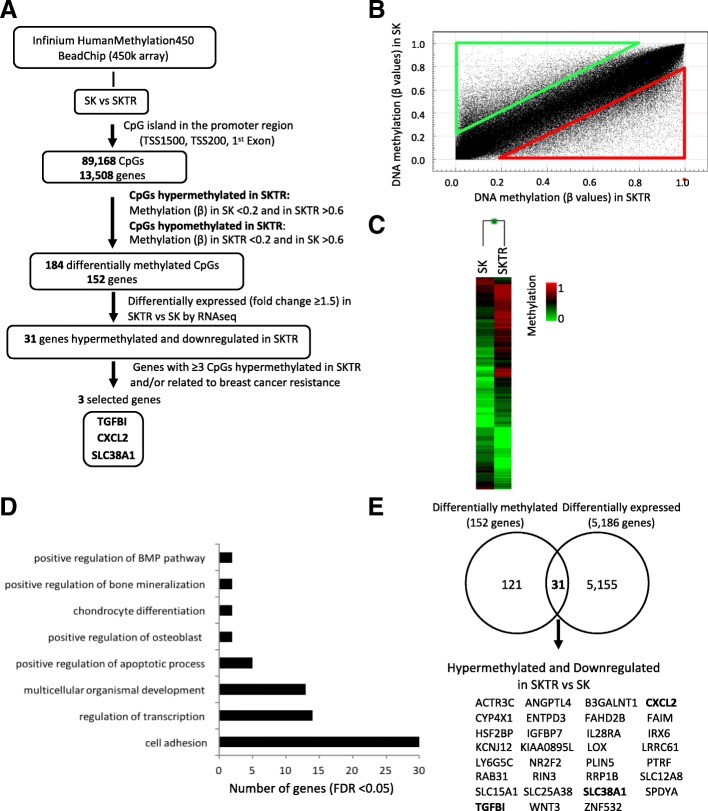
Analysis of the DNA methylation profile associated to trastuzumab resistance in breast cancer cell lines. **a** Schematic flow chart used to identify differentially methylated genes associated to trastuzumab resistance after comparing the sensitive (SK) and resistant (SKTR) to trastuzumab human breast cancer cell lines. **b** Scatter plot representing DNA methylation normalized levels (*β* values) of SK and SKTR cell lines. Red and green triangles indicate the CpGs that gained and lost, respectively, a methylation level ≥ 0.20 in SKTR with respect to SK cells. **c** Supervised hierarchical clustering of the most variable CpGs (Δ*β* ≥ 0.20) from island and promoter regions between the SK and SKTR cell lines. **d** Summary of gene ontology (GO) analysis of the biological process categories for the 152 differentially methylated genes at CpG island and promoter levels between the SK and SKTR cell lines. **e** Venn diagram showing the differentially methylated and differentially expressed genes (obtained by RNA-Seq) between the SK and SKTR cell lines. The name of the 31 hypermethylated and downregulated genes in SKTR is indicated

The Infinium HumanMethylation 450 BeadChip (450k array) is a validated tool for carrying out epigenomic projects because it allows the methylation status of approximately 450,000 CpGs located throughout the human genome to be detected [20]. Our group recently employed this approach in previous studies aimed at identifying genes regulated by DNA methylation for clinical applications such as biomarkers [23, 24]. Therefore, we took advantage of the 450K array methodology to characterize the DNA methylation profile associated with trastuzumab resistance in BC, and compared a trastuzumab-sensitive (SK) and trastuzumab-resistant (SKTR) BC models (Fig. 1a). The global analysis of the DNA methylation (*β* values) corresponding to the CpG sites with *p* value < 0.01 (469,927 CpGs) showed differences in the scatter plot (Fig. 1b) between SK and SKTR models (*r*^2^ = 0.93) and revealed 27,314 differentially methylated CpGs (Δ*β* ≥ 0.20) between both models. In particular, the number of CpGs that gained (red triangle in scatter plot) and lost (green triangle in scatter plot) a methylation level ≥ 0.20 in SKTR with respect to SK was 14,845 and 12,469, respectively.

Therefore, we focused our study on analyzing the methylation levels of the CpG sites located at the regulatory regions corresponding to 89,168 CpGs and 13,508 genes. The supervised hierarchical clustering of the most variable CpGs from promoters and islands (Δ*β* ≥ 0.20) showed a methylation pattern that clearly discriminated between the SK and SKTR models (Fig. 1c). Next, we used more stringent criteria to determine the most differentially methylated CpGs in the promoters and islands, considering in SKTR the CpGs with a methylation level (*β*) in SK < 0.20 and in SKTR > 0.60 as hypermethylated, and in SKTR the CpGs with a methylation level (*β*) in SKTR < 0.20 and in SK > 0.60 as hypomethylated. This analysis revealed 184 differentially methylated CpGs corresponding to 152 genes which, according to a Gene Ontology analysis (GO), were significantly associated (FDR < 0.05) with several biological processes related to cancer, such as cell adhesion pathways (GO:0007155), regulation of transcription (GO:0006355), development (GO:0007275), and control of apoptosis (GO:0043065) (Fig. 1d). To verify whether the methylation observed in these genes had an impact on their transcriptional expression or not, we performed a transcriptomic analysis with RNA-Seq comparing the SK and SKTR models and obtained 1995 overexpressed and 3191 downregulated genes in SKTR displaying a fold change ≥ 1.5 over the SK model. Then, we correlated the genes differentially methylated at the promoter and island with those differentially expressed between SK and SKTR (Fig. 1e). We identified 31 hypermethylated and downregulated genes and no gene hypomethylated and overexpressed in SKTR with respect to the SK model (Table 1). From this list of 31 genes, we selected three genes: *TGFBI* (transforming growth factor beta induced), CXCL*2* (C-X-C motif chemokine ligand 2), and *SLC38A1* (Solute carrier Family 38 Member 1) for further analysis. *TGFBI*, *CXCL2*, and *SLC38A1* were selected because of the high number of differentially methylated CpGs (≥ 3 CpG sites) they presented and their previous implications in BC [25–27].

**Table 1:** Thirty-one differentially methylated and differentially expressed genes between the SK and SKTR models

TargetID	Chr	Position	Gene name	Gene region	SK	SKTR	SKTR vs SK
cg08757148	1	24513722	IL28RA;IL28RA;IL28RA	1stExon;1stExon;1stExon	0.11	0.67	0.56
cg03470088	1	24513939	IL28RA;IL28RA;IL28RA	TSS200;TSS200;TSS200	0.05	0.79	0.75
cg26558485	1	47489282	CYP4X1;CYP4X1	1stExon;5′UTR	0.14	0.69	0.54
cg06816106	2	29033352	SPDYA;SPDYA	TSS1500;TSS1500	0.18	0.64	0.46
cg14798656	2	97760745	FAHD2B	TSS200	0.17	0.62	0.45
cg25999267	3	39424992	SLC25A38;SLC25A38	1stExon;5′UTR	0.19	0.75	0.56
cg17264618	3	40429014	ENTPD3	5′UTR	0.14	0.61	0.46
cg09363539	3	124931746	SLC12A8	TSS200	0.01	0.82	0.81
cg15860013	3	138327718	FAIM;FAIM;FAIM;FAIM;FAIM;FAIM;FAIM	5′UTR;TSS200;1stExon;5′UTR;1stExon;5′UTR;1stExon	0.18	0.68	0.50
cg20841906	3	160822911	B3GALNT1;B3GALNT1;B3GALNT1;B3GALNT1;B3GALNT1	5′UTR;5′UTR;5′UTR;5′UTR;TSS1500	0.06	0.70	0.64
cg20986370	4	57976171	IGFBP7	1stExon	0.19	0.69	0.49
cg19031658	4	74964856	*CXCL2;CXCL2*	1stExon;5′UTR	0.05	0.89	0.85
cg22847221	4	74964920	*CXCL2;CXCL2*	1stExon;5′UTR	0.13	0.61	0.48
cg00630212	4	74965135	*CXCL2*	TSS200	0.16	0.65	0.49
cg18804985	4	74965226	*CXCL2*	TSS1500	0.18	0.96	0.78
cg01429321	5	121413797	LOX;LOX	5′UTR;1stExon	0.10	0.62	0.52
cg21034676	5	135364552	*TGFBI*	TSS200	0.11	0.61	0.51
cg14120129	5	135364575	*TGFBI*	TSS200	0.06	0.61	0.55
cg09873933	5	135364580	*TGFBI*	TSS200	0.09	0.63	0.54
cg07151644	6	31649089	LY6G5C	TSS1500	0.16	0.60	0.44
cg07753583	7	150020206	LRRC61;ACTR3C;LRRC61	TSS200;5′UTR;TSS200	0.17	0.86	0.69
cg10348193	7	150020240	LRRC61;ACTR3C;LRRC61	TSS200;5′UTR;TSS200	0.15	0.97	0.82
cg11026333	7	150020269	LRRC61;ACTR3C;LRRC61	TSS200;5′UTR;TSS200	0.06	0.98	0.92
cg01270001	7	150020401	LRRC61;ACTR3C;LRRC61;LRRC61;LRRC61	1stExon;5′UTR;5′UTR;5′UTR;1stExon	0.13	0.94	0.81
cg22893248	7	150020751	ACTR3C;ACTR3C;LRRC61;LRRC61	1stExon;5′UTR;5′UTR;5′UTR	0.19	0.73	0.54
cg09327770	12	46663270	*SLC38A1;SLC38A1*	TSS200;TSS200	0.02	0.83	0.81
cg20463033	12	46663274	*SLC38A1;SLC38A1*	TSS200;TSS200	0.01	0.77	0.76
cg24795297	12	46663281	*SLC38A1;SLC38A1*	TSS200;TSS200	0.07	0.77	0.71
cg03859162	13	99404887	SLC15A1;SLC15A1	1stExon;5′UTR	0.12	0.66	0.55
cg03485262	14	92980031	RIN3	TSS200	0.04	0.72	0.68
cg08114373	14	92980204	RIN3;RIN3	5′UTR;1stExon	0.17	0.61	0.44
cg18614734	15	96876248	NR2F2;NR2F2;NR2F2;MIR1469;NR2F2	Body;Body;5′UTR;TSS1500;TSS1500	0.18	0.65	0.47
cg01549404	16	55358636	IRX6;IRX6	5′UTR;1stExon	0.11	0.61	0.50
cg01568244	16	67218584	KIAA0895L;EXOC3L	TSS1500;Body	0.14	0.82	0.69
cg01666600	17	21279561	KCNJ12	TSS200	0.05	0.70	0.65
cg03928539	17	21279613	KCNJ12	TSS200	0.08	0.62	0.54
cg01637175	17	21281507	KCNJ12	5′UTR	0.08	0.78	0.70
cg11804833	17	40575289	PTRF;PTRF	1stExon;5′UTR	0.08	0.62	0.54
cg06093379	17	44896080	WNT3;WNT3	5′UTR;1stExon	0.01	0.83	0.82
cg24441185	18	9708096	RAB31	TSS200	0.06	0.92	0.86
cg17289202	18	56530789	ZNF532	5′UTR	0.13	0.80	0.67
cg22932336	19	4535070	PLIN5	5′UTR	0.10	0.88	0.78
cg04532834	19	4535188	PLIN5;PLIN5	1stExon;5′UTR	0.17	0.64	0.46
cg02505409	19	8429160	ANGPTL4;ANGPTL4;ANGPTL4;ANGPTL4	5′UTR;1stExon;5′UTR;1stExon	0.16	0.79	0.63
cg06837791	19	8429491	ANGPTL4;ANGPTL4	1stExon;1stExon	0.17	0.61	0.44
cg12831261	21	45078437	RRP1B;HSF2BP	TSS1500;5′UTR	0.01	0.93	0.92

*Italics denotes the three final genes selected for further analysis

### Epigenetic silencing of *TGFBI*, *CXCL2*, and *SLC38A1* in trastuzumab-resistant cells (SKTR)

**Fig. 2:**
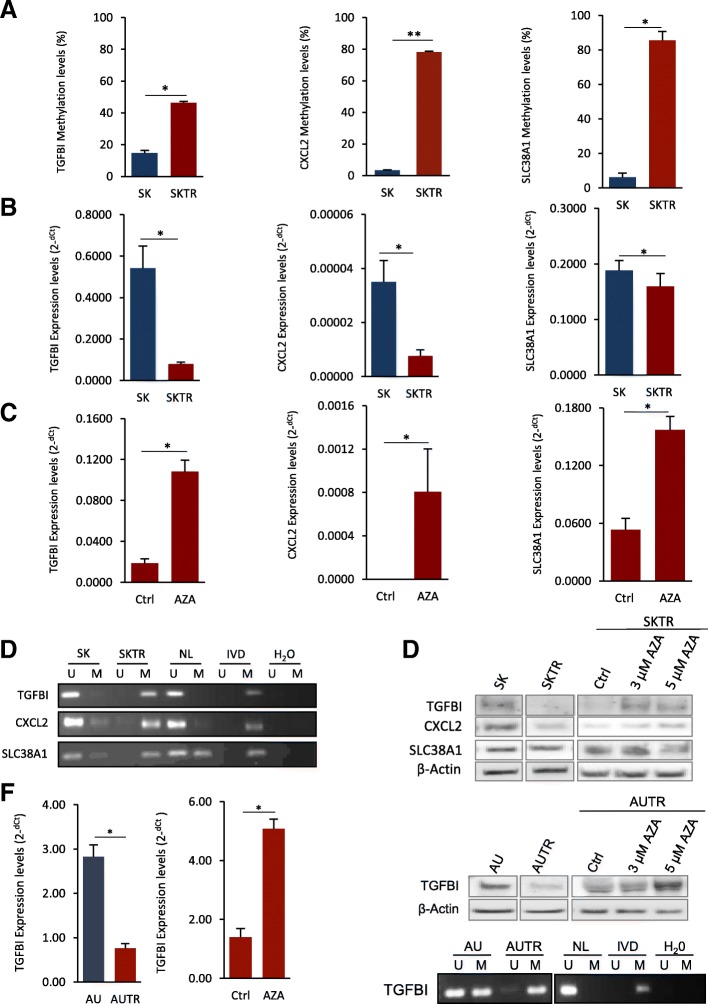
DNA methylation-associated silencing of selected genes comparing the trastuzumab-resistant and trastuzumab-sensitive cell line. **a** DNA methylation levels of *TGFBI*, *CXCL2*, and *SLC38A1* in SK and SKTR cell lines by bisulfite pyrosequencing and **b** methylation-specific polymerase chain reaction (MSP) analysis. **c** Expression levels of *TGFBI*, *CXCL2*, and *SLC38A1* in the unmethylated (SK) and methylated (SKTR) cell lines determined by qRT-PCR. **d** Restored expression of selected genes (*TGFBI*, *CXCL2*, and *SLC38A1*) after DNA demethylating agent 5-aza-2′-deoxycytidine (5-aza-dC) in the SKTR methylated cell line by qRT-PCR. **e** Protein expression of all selected genes (*TGFBI*, *CXCL2*, and *SLC38A1*) in SK and SKTR cells before and after 5-aza-dC treatment by Western blot. **f** Analysis in AU and AUTR cell lines of TGFBI methylation by MSP (top) and (middle) its transcriptional (qRT-PCR) and (bottom) protein levels (Western blot) before and after 5-aza-dC treatment. In MSP, the presence of visible polymerase chain reaction products in lanes marked U indicates unmethylated sequences; the presence of products in lanes marked M indicates methylated sequences. In vitro methylated DNA (IVD) was used as a positive control for methylated sequences. DNA from normal lymphocytes (NL) was used as a negative control for methylated sequences. Results shown are representative of those obtained from three independent experiments, and b-actin was used as a control. Values from pyrosequencing and qRT-PCR were determined from triplicates and are expressed as the mean ± SEM. Significance of Mann-Whitney *U* test, **p < 0.01; *p < 0.05

First, we analyzed the DNA methylation of the selected genes by bisulfite pyrosequencing, which is a technique that allows single genes to be analyzed on a single CpG level [28]. We observed significantly higher methylation levels for the three genes in SKTR relative to the SK (Fig. 2a; *p* < 0.05 for *TGFBI* and *SLC38A1*; *p* < 0.01 for *CXCL2*). Like the previous transcriptomic analysis with RNA-Seq, the SKTR model also showed a significant reduction in the transcript expression levels of *TGFBI*, *CXCL2*, and *SLC38A1* (*p* < 0.05) when compared to the SK cells analyzed by qRT-PCR (Fig. 2b). Interestingly, *TGFBI* was the gene that showed the greatest decrease (fold change = 6.7) in transcriptional levels in the SKTR model. Similar results were obtained by methylation-specific polymerase chain reaction (MSP) analysis (a very specific and sensitive method [29]) that showed higher methylation in SKTR with respect to the SK cells for all the genes evaluated (Fig. 2d). These results confirmed that hypermethylation of the three selected genes at the promoter and CpG island in SKTR cells is associated with reduce transcriptional levels.

In this sense, after treating the SKTR model with the demethylating agent 5-aza-2′-deoxycytidine (5-aza-dC) at 3 μM and 5 μM (Fig. 2c), the transcriptional levels of *TGFBI*, *CXCL2*, and *SLC38A1* were significantly restored (*p* < 0.05), indicating that methylation is an epigenetic mechanism that has a functional role in the transcriptional control of the three genes in question. Importantly, this epigenetic silencing by DNA methylation observed was also confirmed at the protein level in two (*TGFBI* and *CXCL2*) of the three selected genes (Fig. 2e), showing a reduction of the protein levels in SKTR with respect to SK cells that was significantly restored in the hypermethylated SKTR model after the treatment with 5-aza-dC 3 μM and 5 μM. The change observed in the protein levels after the 5-aza-dC treatment was especially drastic in *TGFBI* gene, where the absence of protein expression induced by the promoter hypermethylation in SKTR was completely recovered after the in vitro demethylation.

Taken together, these results suggest that *TGFBI*, an extracellular matrix (ECM) protein whose secretion is induced by transforming growth factor-β (TGF-β), is the most epigenetically regulated gene of the three selected.

Therefore, to verify that the results obtained for these genes were not specific to the SK and SKTR models, we extended their epigenetic analyses to other trastuzumab-sensitive (AU) and trastuzumab-resistant (AUTR) human BC cell models (Fig. 2f and Additional file 2: Figure S1). Likewise, only *TGFBI* gene showed a high correlation between methylation, expression, and protein levels in the AUTR model. With MSP, we observed that hypermethylation of *TGFBI* in AUTR (compared to AU) was associated with a significant decrease (*p* < 0.05) in the transcriptional and protein levels of the gene. Importantly, the transcriptional and protein levels of *TGFBI* gene were both restored in the hypermethylated AUTR model after the treatment with 5-aza-dC 3 μM and 5 μM. These results confirm that the epigenetic silencing of *TGFBI* by DNA methylation is associated to trastuzumab resistance in human BC cells.

### The role of TGFBI expression in trastuzumab resistance cells (SKTR)

We have shown that *TGFBI* promoter hypermethylation is highly associated with the downregulation of its transcript and protein levels in two trastuzumab-resistant models when compared to the corresponding sensitive models. Therefore, we next examined the functional contribution of epigenetic inactivation of the *TGFBI* gene to trastuzumab resistance. We depleted the endogenous *TGFBI* gene expression in the SK model by stable transfection with two different short hairpin RNAs (shTGFBI A and shTGFBI B). We also rescued the TGFBI expression in the *TGFBI*-hypermethylated and the downregulated SKTR models by stable transfection with a plasmid encoding the full-length TGFBI cDNA (TGFBI).

TGFBI is an extracellular matrix (ECM) protein and plays a role in mediating cell adhesion to the ECM, cell proliferation, adhesion, migration, and differentiation through interacting with collagen, fibronectin, laminin, and several integrins [30–32]. These integrin-binding properties of TGFBI have been related to different integrin-binding motifs located in fasciclin-1 domains, including NKDIL, YH18, and EPDIM as well as an Arg-Gly-Asp (RGD) domain [22]. For this reason, we became interested in determining whether this interaction between TGFBI with the ECM and integrins could be involved in the trastuzumab resistance in our models. With this objective in mind, we also expressed a mutated form of TGFBI with four different altered integrin binding motifs (NKDIL, YH18, EPDIM, and RGD), affecting cellular adhesion through the integrin interactions [22]. The transfection and validation protocols were the same as for the overexpression vector.

**Fig. 3:**
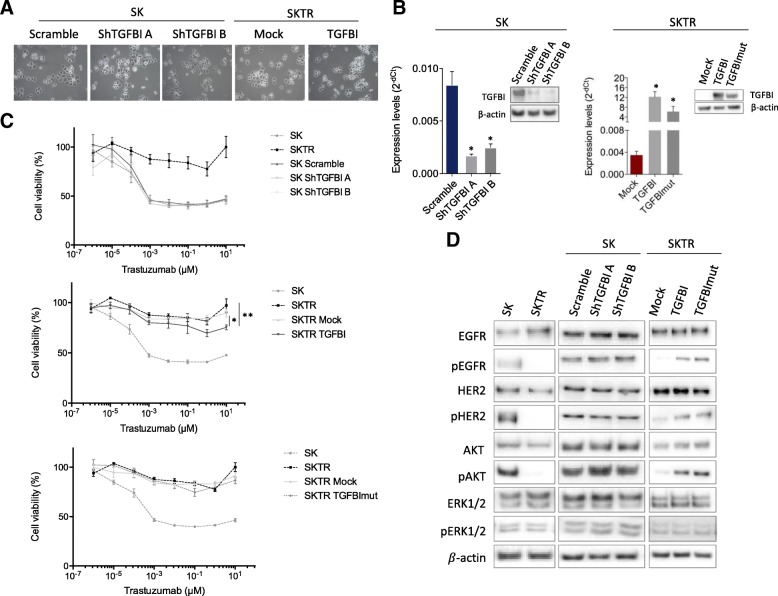
Impact of TGFBI expression in trastuzumab-resistant cells*.* **a** Representative bright-field microscopy images of TGFBI depletion (Scramble, ShTGFBI A, and ShTGFBI B), overexpression (Mock and TGFBI), and mutagenesis (TGFBImut) in SK and SKTR cells. **b** Expression analysis by qRT-PCR and Western blot showing the in vitro stable depletion of TGFBI in SK cells (left) and overexpression or mutagenesis of TGFBI in SKTR cells (right). Values of qRT-PCR were determined from triplicates and are expressed as the mean ± SEM. **c** Cell viability determined by MTT assays upon the use of increasing concentrations of trastuzumab (10^−6^ to 10 μM) for 5 days. (Upper) The TGFBI depletion in SK did not affect cell viability upon trastuzumab treatment. (Middle) The TGFBI overexpression in SKTR cells (TGFBI) give rise to a major sensitivity to trastuzumab at 10 μM. (Bottom) The TGFBI mutagenesis in SKTR cells (TGFBImut) did not affect cell viability after trastuzumab treatment. Results are expressed as percentage of surviving cells after drug treatment (mean ± SEM). One-way ANOVA using a Tukey HSD post hoc test, **p < 0.01; *p < 0.05 indicate levels of statistical significance. **d** HER family receptors and their downstream proteins related to PI3K/AKT and MAPK/ERK1/2 pathway characterization in TGFBI-depletion SK cells and TGFBI-overexpression SKTR cells. Western blot showing an enhanced of phosphorylation levels of HER1, HER2, and AKT upon overexpression and mutagenesis of TGFBI in SKTR cells. TGFBI depletion did not produce any change in the HER receptors and their related downstream proteins. Results shown are representative of those obtained from 3 independent experiments and β-actin was used as a control

Despite its known function in cell adhesion and integrin-mediated signaling, we did not observe morphological changes after depletion, overexpression, or mutagenesis of TGFBI (Fig. 3a). The efficiency of the transfection was assessed by measuring the TGFBI gene expression using quantitative RT-PCR and Western blotting (Fig. 3b). A significant change in its levels was detected both after depletion and overexpression. Upon TGFBI transfection, we analyzed the resistance or sensitivity to trastuzumab in comparison to the parental and empty vector-transfected cells through an MTT assay over 5 days (Fig. 3c; Additional file 3: Figure S2). In the SK model, we observed that TGFBI depletion did not affect the trastuzumab response (Fig. 3c, upper panel). In contrast, overexpression of TGFBI in SKTR led to a significantly higher sensitivity at a trastuzumab concentration of 10 μM compared to the resistant empty vector cell line (Mock: *p* = 0.020) and the SKTR model (*p* = 0.004) (Fig. 3c, middle panel). The TGFBI mutated form (TGFBImut) showed the same response to trastuzumab treatment as the mock vector did, suggesting that TGFBI-mediated trastuzumab sensitivity requires the NKDIL, YH, EPDIM, and RGD binding motifs (Fig. 3c, lower panel).

Therefore, we examined HER family protein receptors and their downstream proteins related to PI3K/AKT and MAPK/ERK1/2 pathways for changes after TGFBI depletion, overexpression, and mutation (Fig. 3d). Consistent with the results of the trastuzumab cell viability, TGFBI depletion in SK cells showed no apparent changes in either total protein or activation levels for any of the proteins examined. Unlike TGFBI depletion, overexpression of TGFBI and TGFBImut in the SKTR model produced changes in the activation levels of some of the HER receptors and downstream signaling proteins in comparison with the mock control vector. In particular, overexpression of either wild type TGFBI or its mutated form resulted in a significant increase in the activation levels of HER1 (pHER1), HER2 (pHER2), and AKT (pAKT) compared to the SKTR model (Mock) that contains hypermethylated *TGFBI* without change in total levels of the respective proteins. Hence, TGFBI overexpression and its mutated form allow the trastuzumab-resistant model to adopt similar activation levels for HER1, HER2, and AKT as the trastuzumab-sensitive cells do.

These data suggest that selective overexpression of TGFBI in the SKTR model (hypermethylated for *TGFBI*) induces an increased sensitivity to trastuzumab and the activation of HER1 and HER2 receptors and the AKT downstream protein. Furthermore, the mutated domains from the second and fourth FAS-1 regions of TGFBI are involved in its response to trastuzumab treatment, but not in its activation or interaction with HER2 downstream proteins. In summary, the SKTR model with an overexpression of TGFBI presents a behavior similar to the SK model.

### *TGFBI* hypermethylation in trastuzumab-resistant HER2+ breast cancer patients

**Fig. 4:**
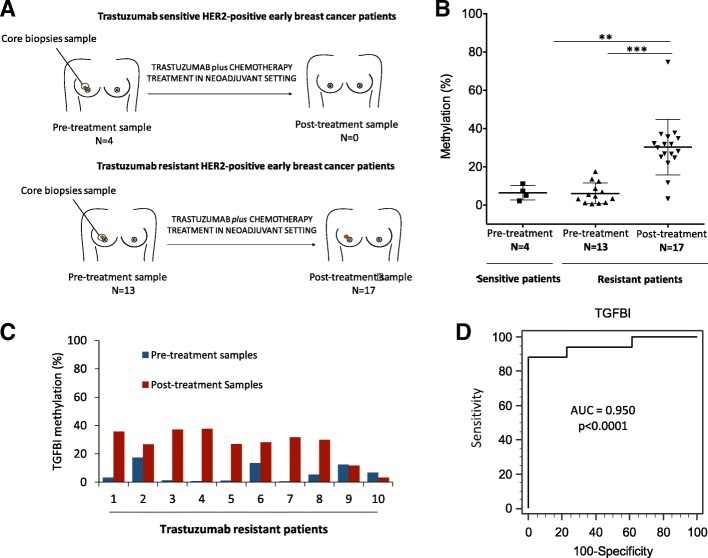
*TGFBI* promoter hypermethylation in HER2+ breast cancer patients with sensitivity and resistance to trastuzumab. **a** Schematic representation of selected patient samples. TGFBI methylation levels were evaluated in tumor samples of 24 HER2+ breast cancer samples. From this cohort, after trastuzumab plus chemotherapy in neoadjuvant regimen, 20 patients developed partial or no response and 4 patients presented complete treatment response. Of the 20 patients with non-response, 10 patients had pre-treatment and post-treatment samples, 3 patients with pre-treatment only samples, and 7 with post-treatment only samples (Top). The 4 patients with complete response to treatment only had pre-treatment samples (Bottom). **b** TGFBI methylation of 3 consecutive CpG sites in the 5′-end promoter CpG island in HER2+ breast cancer treated with trastuzumab analyzed by bisulfite pyrosequencing. The central solid line indicates the median and the limits of the vertical lines show the upper and lower percentiles. **c** TGFBI methylation of 3 consecutive CpG sites in the 5′-end promoter CpG island in resistant patients with paired pre- and post-treatment samples analyzed by bisulfite pyrosequencing. **d** Diagnostic accuracy of TGFBI hypermethylation for resistant HER2+ breast cancer samples. ROC analysis was applied to the TGFBI methylation levels analyzed by pyrosequencing for trastuzumab-resistant samples (pre- and post-treatment samples). Area under the curve (AUC) was 0.9502 (*p* < 0.0001). TGFBI showed great potential for monitoring trastuzumab response in HER2+ breast cancer patients. Significance of Mann-Whitney *U* test is indicated as ***p < 0.001; **p < 0.01

Given the previous in vitro results obtained, we wanted to determine whether the presence of the *TGFBI* promoter CpG island hypermethylation-associated to trastuzumab resistance also occurring in BC patients. Therefore, we evaluated *TGFBI* gene methylation in human tumors from 24 HER2+ early BC patients as a preliminary study. As with our sensitive and resistant models, we analyzed *TGFBI* methylation by bisulfite pyrosequencing in pre-treatment samples from patients who were complete responders and non-responders to trastuzumab-based therapy (Fig. 4a). With the non-responder patients, we also evaluated *TGFBI* methylation after treatment (post-treatment samples), i.e., as we did in our SKTR model. For the non-response patients, we obtained paired samples (pre-treatment and post-treatment samples) from 10 patients, pre-treatment samples only from 3 patients, and post-treatment samples only from 7 patients. For the patients with pathological complete response, only pre-treatment samples were evaluated. The limited number of samples obtained was, in part, due to the exhaustion of the sample during diagnostic procedures and the poor preservation of the DNA in the paraffin tissue (FFPE) blocks.

Our results showed similar pre-treatment *TGFBI* methylation levels (*p* = 0.651) in the patients with complete response to trastuzumab (6.45% ± 1.90) and in the non-responders (6.08% ± 1.51; Fig. 4b). These results indicate that the *TGFBI* methylation levels of the pre-treatment samples are not associated with the absence of response to trastuzumab. In contrast, the non-responsive patients showed significantly higher (*p* = 0.001) methylation levels of *TGFBI* in tumors following treatment with trastuzumab (30.26% ± 3.52) than before starting the therapy (6.08% ± 1.51), suggesting that acquired resistance to trastuzumab is associated with increased methylation levels of *TGFBI*. In particular, when considering the non-responsive patients with pre- and post-treatment paired samples, we observed a significant (*p* < 0.001) *TGFBI* promoter hypermethylation after trastuzumab (8 out of 10 = 80%) compared to the pre-treatment samples, indicating that the increase in *TGFBI* methylation levels is associated with trastuzumab resistance (Fig. 4c). Importantly, the ROC curve analysis with an AUC of 0.95 (*p* < 0.0001; 95% CI 0.803 to 0.996) allowed us to clearly differentiate the methylation levels of *TGFBI* between the pre- and post-treatment samples of the non-responsive patients. This result suggests that *TGFBI* could be a potential biomarker for monitoring trastuzumab response during HER2+ BC patients’ treatment (Fig. 4d). In addition, the Hosmer-Lemeshow test (chi-square = 89,606; *p* = 0.3456) suggests that the methylation of *TGFBI* has a certain ability to predict the resistance to trastuzumab when compared with an ideal model (Additional file 4: Figure S3). On the other hand, no significant association between *TGFBI* methylation levels before and after treatment and the clinical-histopathological characteristics was identified (Table 2).

**Table 2:** Clinical and pathological characteristics in HER2-positive early breast cancer according to *TGFBI* promoter methylation levels

Characteristics	% *N* = 10		*p* value*
	*n*	Median (p25–p75)	
Age	10	−0.383*	0.275*^1^
Menopause			0.151*^2^
Premenopausal	5	32.13 (24.58–35.86)	
Postmenopausal	5	14.58 (−0.68–25.77)	
ER			0.089*^2^
Negative	2	2.85 (−3.57–9.28)	
Positive	8	28.50 (19.58–34.00)	
HER2 status			–
FISH	0	–	
IHC+	10	25.18 (9.28–32.13)	
Histological grade			–
Grade 1–2	10	25.18 (9.28–32.13)	
Grade 3	0	–	
Clinical stage			0.804*^3^
IIB	2	16.15 (−3.57–25.86)	
IIIA	2	19.58 (14.58–24.58)	
IIIB	6	28.50 (9.28–32.13)	
Pathological response			–
No response	1	9.28	
Partial response	9	25.77 (14.58–32.13)	
Percentage of tumor shrinkage**	10	0.029	0.957*^1^
Miller & Payne			1.000*^2^
G2	1	0.68	
G3	2	22.57 (9.28–35.86)	
G4	7	25.77 (19.58–31.68)	
Type of surgery			0.267*^2^
Lumpectomy	2	10.51 (−3.57–24.58)	
Mastectomy	8	28.50 (11.93–34.00)	

*^1^Spearman correlation

*^2^Mann-Whitney test

*^3^Kruskal-Wallis test

## Discussion

HER2 is overexpressed in 20–30% of BC and is associated with aggressive phenotype and poor prognosis. Despite the initial good response to the treatment, a large portion of patients present de novo or acquired treatment resistance. Currently, HER2 detection is the only validated biomarker for predicting the benefit of anti-HER2 therapies [33]. While different cellular and molecular mechanisms involved in trastuzumab resistance have been described, none of them are used to detect, predict, or monitor BC treatment in a clinical routine [7, 8, 34]. Thus, there is great interest in finding potential biomarkers able to detect, predict, or monitor trastuzumab response which, in turn, would allow us to stratify patients and save costs and toxicities for those who do not respond to treatment.

Epigenetic biomarker studies are focused on analyzing the methylation changes in the promoter regions of candidate genes in specific tumor types for the implication in gene silencing [9, 34]. In general, this gene repression results in an adaptive advantage for the cells, allowing a more aggressive and invasive phenotype to be adopted. After analyzing the promoter methylation profile of long-term trastuzumab-sensitive and trastuzumab-resistant models developed in our laboratory [16, 17], three different methylated genes (*TGFBI*, *CXCL2*, and *SLC38A1*) implicated in cancer were identified and subsequently validated using different methylation and expression approaches. Additionally, the selected genes were validated in two other long-term trastuzumab-sensitive (AU) and trastuzumab-resistant (AUTR) human HER2+ BC cell lines developed by our group [17]. Although both cell lines analyzed derive from the same patient and have similar genetic and transcriptomic profiles, they have some differences in transcriptional levels [35]. Moreover, SKBr3 and AU565 have been described as having distinct responses to drug treatment depending on culture conditions [36]. From all the genes selected, *TGFBI* (also known as Big-H3 or keratoepithelin) was the only one with a consistent methylation, expression, and protein pattern in both resistant models. This epigenetic silencing of the *TGFBI* gene observed was in line with previous studies of other cancers [37, 38].

As explained before, TGFBI encodes a 68-kDa secretory protein induced by transforming growth factor-β (TGF-β) in human adenocarcinoma cells as well as other human cell types [39]. According to the bibliography, TGFBI protein is composed by 683 amino acids containing a secretory signal (SP) in the *N-*terminal cysteine-rich domain (CRD), and four fasciclin-1 domains (FAS1-1, FAS1-2, FAS1-3, and FAS1-4), which contain several known integrin-binding motifs including NKDIL, YH18, and EPDIM as well as an Arg-Gly-Asp (RGD) domain [22]. TGFBI has been reported to function as an ECM protein to mediate cell adhesion and migration through interacting with collagen, fibronectin, and laminin and several integrins including α1β1, α3β1, αvβ3, and αvβ5 [30–32]. While TGFBI has been reported to be involved in tumorigenesis, its role is not clear. In this paper, we have linked the promoter DNA methylation-associated silencing of *TGFBI* with a possible tumor suppressor function in trastuzumab-resistant models. Several previous reports have indicated that TGFBI plays the role of a tumor suppressor gene in various cancers such as lung, breast, and ovarian [25, 40–42]. However, in other cancers, such as colon or pancreas, TGFBI has been described as having a tumor-promoting function [43–46]. These opposing effects of TGFBI suggest that its expression and function are dependent on cell type [22].

TGFBI promotes cell adhesion by interacting with different integrins, which, in turn, has been shown to play an important role in tumor progression in humans. TGFBI’s inhibition of invasive capacity correlates with a previous analysis by our group, which revealed a greater invasive capacity for SKTR compared to the SK [16]. To determine if TGFBI has a potential role in trastuzumab resistance, different functional studies were carried out. Our results show that TGFBI overexpression in SKTR significantly sensitizes the cells to the treatment and activates some HER2 downstream protein pathways (pAKT, pHER1, and pHER2), adopting a similar pattern to SK. In contrast, TGFBI knockdown in TGFBI endogenous expressed in the SK cells did not produce any effect. A direct interaction between the HER2 receptor and different integrins has been described in multiple reports [47, 48]. Moreover, different integrins have been related to trastuzumab resistance such as β1 or α6β4 [49, 50]. Therefore, TGFBI mutated vector was performed to investigate the role of the RGD motif and the three integrin-binding motifs NKDIL, EPDIM, and YH18 in trastuzumab response [22]. Like TGFBI overexpression, the TGFBI mutated form induced the activation of different HER2 downstream proteins, but there were no differences in trastuzumab treatment response. Therefore, it is likely that the four mutated domains of TGFBI are required for its function in trastuzumab response, which is consistent with other studies that described the involvement of FAS1 or RGD motifs in tumor angiogenesis and tumor growth inhibition, as well as in promoting apoptosis [31, 51–53]. Moreover, it has also been described that TGFBI is involved in mesothelioma progression through the AKT/mTOR pathway which could be related to the HER2 pathway changes observed in TGFBI overexpression and mutagenesis [54]. In summary, this study suggests that TGFBI expression may promote the effectiveness of trastuzumab treatment.

The methylation of *TGFBI* showed certain capacity to predict the resistance to trastuzumab according to the Hosmer-Lemeshow test; however, these results should be interpreted with caution because the number of grouped patients was small. Although more in vitro functional analysis are necessary to elucidate the TGFBI role in trastuzumab resistance, we suggest some hypothetical situations taking into account all TGFBI action mechanisms. It has been described that TGFBI could inhibit cell adhesion to various ECM proteins inhibiting cell proliferation and invasion in neuroblastoma [55]. Moreover, cell adhesion to ECM has been consistently reported as one of the mechanisms used by tumor cells to resist chemotherapy [56]. These observations are in correlation with previous studies by our group, where it is exposed that our SKTR model presented a high significant adhesion capacity to bind to extracellular matrix proteins like fibronectin, collagen I, collagen IV, or laminin I compared to SK [16]. Therefore, we hypothesized that in the absence of TGFBI, the integrins could bind to the ECM providing attachment sites for cells inducing more invasion, or acting as a physical drug barrier, restricting drug transport and limiting their efficacy. The intratumoral diffusion and physical masking of HER receptors by ECM proteins have also been described as a mechanism that can affect the therapeutic efficacy of some drugs including trastuzumab [57].

Finally, we correlated the previous in vitro results in human samples treated with neoadjuvant anthracycline-taxane-based chemotherapy *plus* trastuzumab. In our preliminary study, a higher *TGFBI* methylation level after treatment was observed in patients who developed treatment resistance. These results are in accordance with other studies which demonstrated an association between *TGFBI* hypermethylation and poor prognosis in prostate and lung cancer [41]. Moreover, decreased *TGFBI* expression was identified in advanced stages of BC and NSCLC tumors [40, 58]. Due to the small size of the patient cohort and the retrospective design, the role of *TGFBI* methylation as a biomarker requires further validation in a larger and independent cohort. Nevertheless, the present study brings to light for the first time that *TGFBI* methylation has a significant discriminative value between pre- and post-treatment samples, as demonstrated by ROC curve analyses. Interestingly, the *TGFBI* promoter methylation analysis in responder patients showed similar methylation levels with pre-treatment samples of non-responders. This observation suggests a possible role of *TGFBI* as a monitoring biomarker for trastuzumab response in patients with HER2+ BC.

Although no significant association was found between *TGFBI* methylation before and after treatment and patients’ clinical-histopathologic characteristics, a high number ER-positive patients were observed. In this sense, previous studies have shown that there is crosstalk between ER and HER2 pathways and it affects the response to treatment [59]. In addition, although the type of surgery is not associated with a higher *TGFBI* methylation level in post-treatment samples, 73% of our patients were treated with mastectomy versus 27% with lumpectomy, probably due to the high prevalence of stage III. The low histological grade and high clinical stage to treatment were probably due to most of the patients having non-operable cancers including inflammatory tumors. Currently, neoadjuvant treatment is generally used for operable HER2+ BC thanks to an improvement in the efficacy of the drugs used for treatment [60]. At the time our cohort was treated, none of these drugs were yet available. That said, trastuzumab remains the gold standard treatment for HER2+ BC.

## Conclusions

The results obtained in this study have provided an overview of the DNA methylation pattern in HER2+-resistant BC. *TGFBI* promoter hypermethylation could be a potential methylation monitoring biomarker for trastuzumab response. In addition, once the resistance is developed, demethylation of *TGFBI* with compounds that inhibit DNA methyltransferases could contribute to the sensitization of breast cancer cells to the trastuzumab treatment. However, further studies are required to identify the specific role *TGFBI* plays in trastuzumab resistance in the neoadjuvant settings to definitively show *TGFBI* as a clinical relevant biomarker. Future studies based on *TGFBI* promoter methylation analyses in circulating DNA during neoadjuvant treatment could be a potential strategy with which to confirm our findings.

## Additional files

Additional file 1: Additional methods. (DOCX 30 kb)

Additional file 2: ** Figure S1.** DNA methylation-associated silencing of *CXCL2* and *SLC38A1* comparing the trastuzumab-resistant (AUTR) and -sensitive (AU) AU565 cell model. (A) Expression levels of *CXCL2* and *SLC38A1* in the unmethylated (AU) and methylated (AUTR) models determined by qRT-PCR. (B) Restored expression of *CXCL2* and *SLC38A1* after DNA demethylating agent 5-aza-2′-deoxycytidine (5-aza-dC) in the AUTR methylated cell line by qRT-PCR. (C) DNA methylation levels of *CXCL2* and *SLC38A1* in AU and AUTR cell lines by methylation-specific polymerase chain reaction (MSP) analysis. (D) Protein expression of *CXCL2* and *SLC38A1* in AU and AUTR cells before and after 5-aza-dC treatment by Western blot. In MSP, the presence of visible polymerase chain reaction products in lanes marked U indicate unmethylated sequences; the presence of products in lanes marked M indicate methylated sequences. In vitro methylated DNA (IVD) was used as a positive control for methylated sequences. DNA from normal lymphocytes (NL) was used as a negative control for methylated sequences. Results shown are representative of those obtained from three independent experiments and b- actin was used as a control. Values from pyrosequencing and qRT-PCR were determined from triplicates and are expressed as the mean ± SEM. Significance of Mann-Whitney *U* test, **p < 0.01; *p < 0.05. (PDF 235 kb)

Additional file 3: ** Figure S2.** Cell viability determined by MTT assays upon trastuzumab treatment for 5 days. (A) TGFBI depletion in SK cells, (B) TGFBI overexpression in SKTR cells (TGFBI) and (C) TGFBI-mutagenesis in SKTR cells (TGFBImut). The continuous line represents the fitted dose-response curve while discontinuous lines indicate the simultaneous 95% confidence interval (CIs) for the continuous line with the same color. ANOVA using a Tukey HSD post hoc test, **P < 0.01; *P < 0.05 indicate levels of statistical significance. (PDF 397 kb)

Additional file 4: **Figure S3.** Calibration curve for the reliability of TGFBI to predict trastuzumab resistance. Calibration was assessed using the Hosmer-Lemeshow test and graphically using a calibration plot. The observed frequency was calculated for each group. Predicted probability refers to the predicted probabilities generated by the model. Patients were grouped according to deciles of the predicted probabilities. Grouped patients refers to patients grouped at each decile of predicted probabilities generated by the model. A LOWESS regression was fit to all data points (blue line). (PDF 119 kb)

## Data Availability

The microarray and sequencing dataset generated during the current study is available in NCBI GEO (www.ncbi.nlm.nih.gov/geo) under accession GSE123754 and GSE114575, respectively.

## Abbreviations

5-aza-dC: 5-Aza-2′-deoxycytidine
Akt: Protein kinase B
BC: Breast cancer
CXCL2: C-X-C motif chemokine ligand 2
ECM: Extracellular matrix
FDR: False discovery rate
GO: Gene ontology
HER: Human epidermal growth factor receptor family
HER2: Human epidermal growth factor receptor 2
HER2+: HER2-positive
MAPK: Ras-mitogen-activated protein kinases
MSP: Methylation-specific PCR
PI3K: Phosphatidylinositol 3′-kinase
SLC38A1: Solute carrier Family 38 Member 1
TGF-β: Transforming growth factor beta
TGFBI: Transforming growth factor beta induced
