# Supplementary material for: Epigenetic silencing of TGFBI confers resistance to trastuzumab in human breast cancer
Source: Breast Cancer Res. 2019 Jul 5;21:79. doi: 10.1186/s13058-019-1160-x (PMC6612099; doi:10.1186/s13058-019-1160-x)
Supplement: Supplementary file 1 — Additional methods. (DOCX 30 kb) [file 13058_2019_1160_MOESM1_ESM.docx]

**Additional file 1: Additional methods**

**RNA sequencing**

Illumina TruSeq Stranded mRNA Sample Prep with oligo dT selection 3.1. Library construction is performed using the Illumina TruSeq Stranded mRNA Sample Preparation Kit (cat# RS-122-2101, RS-122-2102). Briefly, total RNA (100 ng to 4 ug) is poly-A selected using poly-T oligo-attached magnetic beads. Poly-A RNA eluted from the magnetic beads is fragmented and primed with random hexamers in preparation for cDNA synthesis. First strand reverse transcription is accomplished using Superscript II Reverse Transcriptase (Invitrogen cat#18064-014). Second strand cDNA synthesis is accomplished using DNA polymerase I and Rnase H under conditions in which dUTP is substituted for dTTP, yielding blunt-ended cDNA fragments in which the second strand with dUTP. An A-base is added to the blunt ends as a means to prepare the cDNA fragments for adapter ligation and block concatamer formation during the ligation step. Adapters containing a T-base overhang are ligated to the A-tailed DNA fragments. Ligated fragments are PCR-amplified (12-15 cycles) under conditions in which the PCR reaction enables amplification of the first strand cDNA product, whereas attempted amplification of the second strand product stalls at dUTP bases and therefore is not represented in the amplified library. The PCR-amplified library is purified using Agencourt AMPure XP beads (Beckman Coulter Genomics cat#A63881). Following amplification, the library is purified by bead based methodologies. The concentration of the amplified library is measured with a NanoDrop spectrophotometer and an aliquot of the library is resolved on an Agilent 2200 Tape Station using a D1K (cat# 5067-5361 and 5067-5362) or a High Sensitivity D1K (cat# 5067-5363 and 5067-5364) assay to define the size distribution of the sequencing library. Libraries are adjusted to a concentration of approximately 10nM and quantitative PCR is performed using the KapaBiosystems Kapa Library Quant Kit (cat# KK4824) to calculate the molarity of adapter ligated library molecules. The concentration is further adjusted following qPCR to prepare the library for Illumina sequence analysis using HiSeq 101 Cycle paired-end sequencing. Sequencing libraries (25 pM) were chemically denatured are applied to an Illumina HiSeq v4 paired end flow cell using an Illumina cBot.  Hybridized molecules were clonally amplified and annealed to sequencing primers with reagents from an Illumina HiSeq PE Cluster Kit v4-cBot (PE-401-4001).  Following transfer of the flow cell to an Illumina HiSeq instrument, a 101 cycle paired-end sequence run was performed using HiSeq SBS Kit v4 sequencing reagents (FC-401-4002).

**Short hairpin interference and ectopic expression assays**

To achieve a long-term knockdown, two different ShRNAs were specifically designed against TGFBI mRNA (NM_000358) in two different loci, considering a 19-base target sequence. For comparison purposes and to evaluate their knockdown efficiency targeting TGFBI-expressing cells, an ShRNA against MSS2 yeast protein (NM_001180166), absent in mammals, was considered as a Scramble^27^^1^. The shRNA vectors were created by oligonucleotide-based cloning. Briefly, the designed shRNA oligos were annealed and inserted between the BamHI and EcoRI restriction sites in pLVX-shRNA2 vector (Clontech). For shRNA oligonucleotides please refer to Supplementary Table 4. 50 μl of jetPRIME® (Polyplus-transfection S.A., Illkirch, France) was added to a mixture of 10 μg of each ShRNA, 7.5 μg of psPAX2 and 2.5 μg of pMD2.G plasmids in 1 mL of jetPRIME® buffer. Each transfection mix was then vortexed and incubated at RT for 10 min and subsequently added drop-wise to a 10-cm dish containing Lenti-X™ 293T cells (Clontech) at 80% confluence. After culturing the cells for 4 hours, the culture medium was replaced by 10 mL of fresh pre-heated medium (DMEM). Viral supernatants were collected and 0.45-μm filtered at 72 hours. The same procedure was used for ectopic overexpression experiments. TGFBI ORF was amplified from SKWT cDNA using specific primers with end-adaptors containing XhoI and NotI restriction enzyme sites and a Kozak sequence (Supplementary Table 4). These restriction enzymes were used to digest both PCR fragment and pLVX-IRES-tdTomato vector (Clontech) that were subsequently ligated and verified by Sanger-sequencing. This construction (wild-type) was then used as a template to create its mutated version following a PCR based strategy (Supplementary Table 4). Mutations were performed in different binding motifs of the protein, the RGD motif in the C-terminus, the NKDIL motif (amino acids 354-358), the highly conserved tyrosine and histidine residues YH18 motif (amino acids 563-580) and the EPDIM motif (amino acids 617-621) in the second and fourth FAS-1 domains^28^. Lentiviral production was performed following the same procedures as for shRNA constructs. After lentiviral transduction, ZsGreen1-positive or tdTomato-positive cells were sorted by flow cytometry.

**Supplemental tables**

Table 1. Pyrosequencing primers

| CXCL2_**F** | GGTTTTTTAGTTTTAATTATGTATAAAAGG |
| --- | --- |
| CXCL2_**R** | ACCTATAACCCRAACTCTATAACT |
| **PyroSeq_**CXCL2 | AGTTTTAATTATGTATAAAAGGG |
| SLC38A1_**F** | TGTAGGTTGTGTTTAGGGTTAGT |
| SLC38A1_**R** | AAAAAACRAAAAAATTACATTATA |
| **PyroSeq**_SLC38A1 | AAAATAACCRCRAAAATAACAAATC |
| TGFBI_**F** | TGGGTGTTTAGGGTAGTTA |
| TGFBI_**R** | CCCRAACCAAATTAAATAA |
| **PyroSeq**_TGFBI | GGTGTTTAGGGTAGTTAG |

Table 2. MSP primers

|  | **Forward** | **Reverse** |
| --- | --- | --- |
| CXCL2_Methylation | TTAAGGGATTTGATTTACGAC | CGAATCCCTAAAACGAAA |
| CXCL2_Unmethylation | GTTTAAGGGATTTGATTTATGAT | CCCAAATCCCTAAAACAAAA |
| SLC38A1_Methylation | GTTTTTCGGGTTGCGTTCG | TTTTAAATACGCCAAAACCCCG |
| SLC38A1_Unmethylation | GGTTTTTTGGGTTGTGTTTG | ATTTTAAATACACCAAAACCCCA |
| TGFBI_Methylation | AGGGTAGTTAGGGGCGTAC | CCAAATTAAATAAACTACGAACG |
| TGFBI_Unmethylation | TTTAGGGTAGTTAGGGGTGTAT | AAACCAAATTAAATAAACTACAAACA |

Table 3. Primers for qRT-PCR with SYBER Green

|  | **Forward** | **Reverse** |
| --- | --- | --- |
| CXCL2 | gtcatttgttaatatttcttcgtgatg | cactggccattttcttgga |
| SLC38A1 | cttttgccacctttcccttt | gagagaaacaaacatgctccaa |
| TGFBI | GTGTGTGCTGTGCAGAAGGT | CATATCCAGGACAGCACTCG |
| GAPDH | ATCATCCCTGCCTCTACTGG | GTCAGGTCCACCACTGACAC |

Table 4. Oligonucleotides used to create the lentiviral vector

| **Name** | **Sequence** |
| --- | --- |
| Scramble-F | gatccGCGCAGAACAAATTCGTCCATTCAAGAGATGGACGAATTTGTTCTGCGTTTTTTacgcgtg |
| Scramble-R | aattcacgcgtAAAAAACGCAGAACAAATTCGTCCATCTCTTGAATGGACGAATTTGTTCTGCGCg |
| TGFBI-Sh1-S | gatccGGTTATTGGCACTAATAGGTTCAAGAGACCTATTAGTGCCAATAACCTTTTTTg |
| TGFBI-Sh1-AS | aattcAAAAAAGGTTATTGGCACTAATAGGTCTCTTGAACCTATTAGTGCCAATAACCg |
| TGFBI-Sh2-S | gatccGCACGATGCTTGAAGGTAACTTCAAGAGAGTTACCTTCAAGCATCGTGTTTTTTg |
| TGFBI-Sh2-AS | aattcAAAAAACACGATGCTTGAAGGTAACTCTCTTGAAGTTACCTTCAAGCATCGTGCg |
| TGFBI- Sh3-S | gatccGTGGCGTGGTCCATGTCATCTTCAAGAGAGATGACATGGACCACGCCATTTTTTg |
| TGFBI- Sh3-AS | aattcAAAAAATGGCGTGGTCCATGTCATCTCTCTTGAAGATGACATGGACCACGCCACg |
| TGFBI-Sh4-S | gatccGCAGTCATCAGCTACGAGTGTTCAAGAGACACTCGTAGCTGATGACTGTTTTTTg |
| TGFBI-Sh4-AS | aattcAAAAAACAGTCATCAGCTACGAGTGTCTCTTGAACACTCGTAGCTGATGACTGCg |
| TGFBI-Sh5-S | gatccGGAAGGCGATCATCTCCAATTCAAGAGATTGGAGATGATCGCCTTCCTTTTTTg |
| TGFBI-Sh5-AS | aattcAAAAAAGGAAGGCGATCATCTCCAATCTCTTGAATTGGAGATGATCGCCTTCCg |
| TGFBI-XhoI-S | aaaaaaCTCGAGCCGCCACCATGGCGCTCTTCGTGCGGCTGC |
| TGFBI-mut1-AS | GTGGCTAGGATAATAATAATGGAGATGATCGCCTTCCCGTTGATAGTG |
| TGFBI-mut1-S | GATCATCTCCATTATTATTATCCTAGCCACCAACGGGGTGATCCACTACATTGATGAGC |
| TGFBI-mut2-AS | CATCACCAATGAAGAATTTCAGGATGTTGGCAAGTTCCTTGGCATC |
| TGFBI-mut2-S | CATCCTGAAATTCTTCATTGGTGATGAAATCCTGGTTAGCGGAGGCATCGGGG |
| TGFBI-mut3-AS | GTGGCCATGATAATAGGAATGGCAACAGGCTCCTTGTTGACACTCACCACATTGTTTTTC |
| TGFBI-mut3-S | GAGCCTGTTGCCATTCCTATTATCATGGCCACAAATGGCGTGGTCCATGTCATCACCAATGTT |
| TGFBI-mut4-AS | CTGCAAGTTCAATGGCAATTTCCTGAGGTCTGTTGGCTGGAGGCTGCAGAACATTG |
| TGFBI-mut4-S | GACCTCAGGAAATTGCCATTGAACTTGCAGACTCTGCGCTTGAGATCTTCAAACAAG |
| TGFBI-NotI-AS | aaaaaaGCGGCCGCCTAATGCTTCATCCTCTCTAATAACTTTTGATAGACAGGGG |
| TGFBI-FLAG-NotI-AS | aaaaaaGCGGCCGCCTACTTgTCGTCGTCgTCCTTGTAgTCgCCgctgCCATGCTTCATCCTCTCTAATAACTTTTGATAGACAGGGGC |

Table 5. Antibody description

| **Antibody** | **#Ref** | **Supplier** | **Dilution** | **Source** |
| --- | --- | --- | --- | --- |
| **CXCL2** | ab9841 | Abcam | 1:500 | Rabbit |
| **SLC38A1** | ab60145 | Abcam | 1:200 | Rabbit |
| **TGFBI** | BAF2935 | R&D System | 1:500 | Goat |
| **EGFR** | 2231 | Cell Signaling Technology | 1:1000 | Rabbit |
| **p-EGFR** | 2234 | Cell Signaling Technology | 1:500 | Rabbit |
| **HER2** | 2165 | Cell Signaling Technology | 1:1000 | Rabbit |
| **p-HER2** | 6942 | Cell Signaling Technology | 1:500 | Rabbit |
| **AKT** | 9272 | Cell Signaling Technology | 1:1000 | Rabbit |
| **p-AKT** | 4058 | Cell Signaling Technology | 1:1000 | Rabbit |
| **ERK1/2** | 9102 | Cell Signaling Technology | 1:1000 | Rabbit |
| **p-ERK1/2** | 9106 | Cell Signaling Technology | 1:1000 | Rabbit |
| **α-tubulin** | 3873 | Cell Signaling Technology | 1:1000 | Mouse |
| **β-actin** | 3700 | Cell Signaling Technology | 1:1000 | Mouse |
