# Supplementary figures and images for: Epigenetic silencing of TGFBI confers resistance to trastuzumab in human breast cancer
Source: Breast Cancer Res. 2019 Jul 5;21:79. doi: 10.1186/s13058-019-1160-x (PMC6612099; doi:10.1186/s13058-019-1160-x)

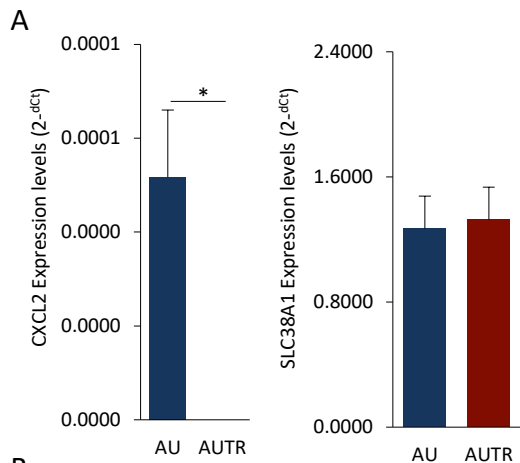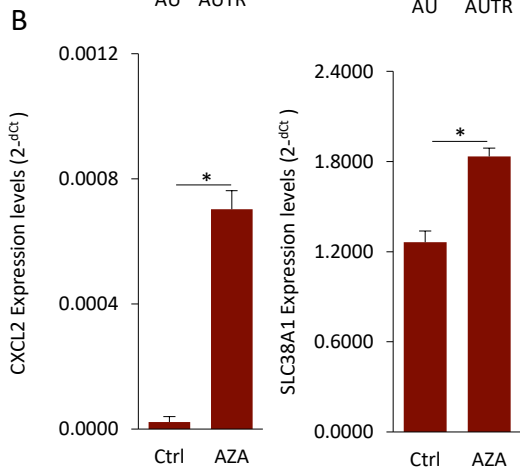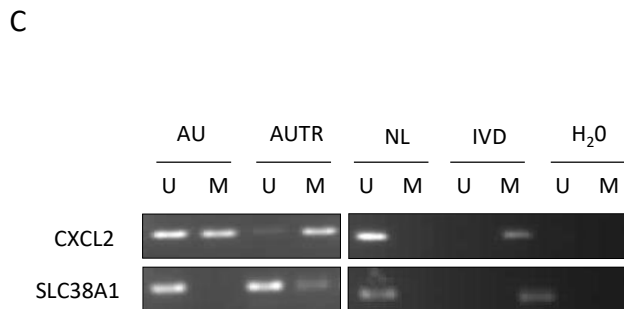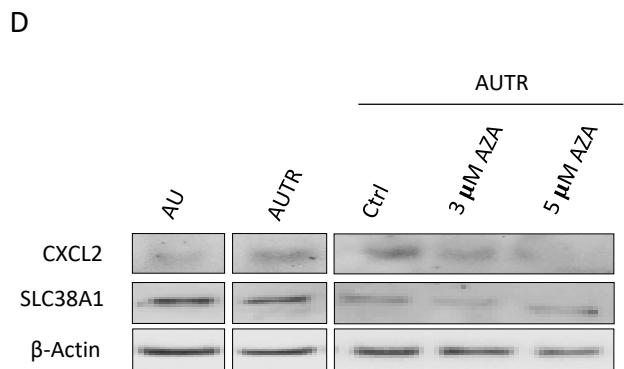

Supplement: Supplementary file 2 — Figure S1. DNA methylation-associated silencing of CXCL2 and SLC38A1 comparing the trastuzumab-resistant (AUTR) and -sensitive (AU) AU565 cell model. (A) Expression levels of CXCL2 and SLC38A1 in the unmethylated (AU) and methylated (AUTR) models determined by qRT-PCR. (B) Restored expression of CXCL2 and SLC38A1 after DNA demethylating agent 5-aza-2′-deoxycytidine (5-aza-dC) in the AUTR methylated cell line by qRT-PCR. (C) DNA methylation levels of CXCL2 and SLC38A1 in AU and AUTR cell lines by methylation-specific polymerase chain reaction (MSP) analysis. (D) Protein expression of CXCL2 and SLC38A1 in AU and AUTR cells before and after 5-aza-dC treatment by Western blot. In MSP, the presence of visible polymerase chain reaction products in lanes marked U indicate unmethylated sequences; the presence of products in lanes marked M indicate methylated sequences. In vitro methylated DNA (IVD) was used as a positive control for methylated sequences. DNA from normal lymphocytes (NL) was used as a negative control for methylated sequences. Results shown are representative of those obtained from three independent experiments and b- actin was used as a control. Values from pyrosequencing and qRT-PCR were determined from triplicates and are expressed as the mean ± SEM. Significance of Mann-Whitney U test, **p < 0.01; *p < 0.05. (PDF 235 kb) [file 13058_2019_1160_MOESM2_ESM.pdf]

A

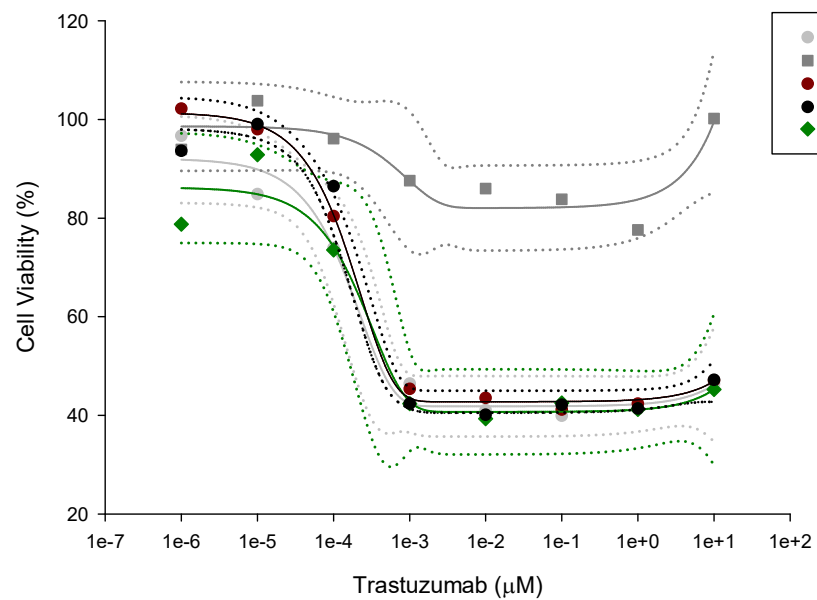

B

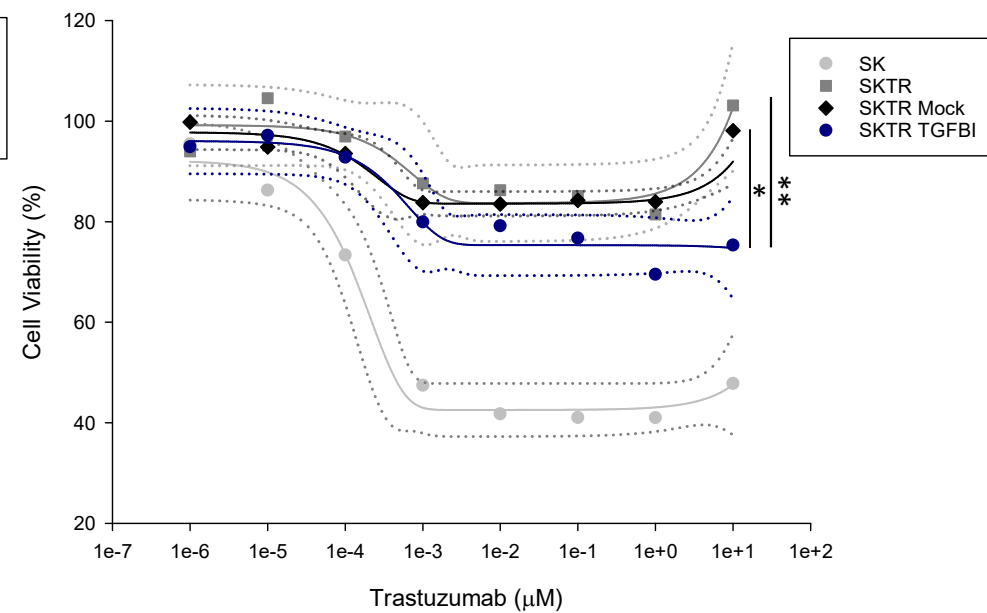

C

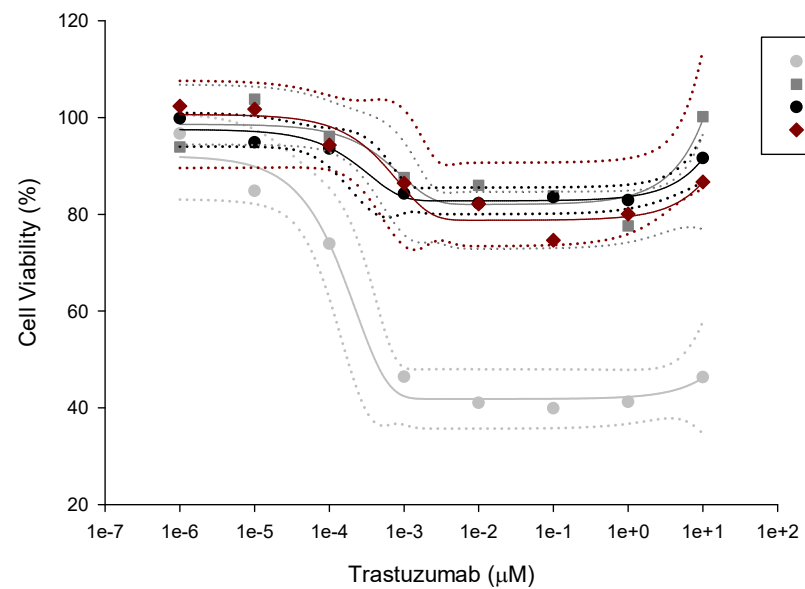

Supplement: Supplementary file 3 — Figure S2. Cell viability determined by MTT assays upon trastuzumab treatment for 5 days. (A) TGFBI depletion in SK cells, (B) TGFBI overexpression in SKTR cells (TGFBI) and (C) TGFBI-mutagenesis in SKTR cells (TGFBImut). The continuous line represents the fitted dose-response curve while discontinuous lines indicate the simultaneous 95% confidence interval (CIs) for the continuous line with the same color. ANOVA using a Tukey HSD post hoc test, **P < 0.01; *P < 0.05 indicate levels of statistical significance. (PDF 397 kb) [file 13058_2019_1160_MOESM3_ESM.pdf]

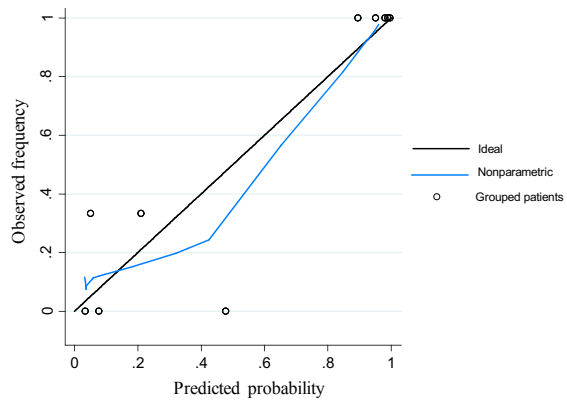

Supplement: Supplementary file 4 — Figure S3. Calibration curve for the reliability of TGFBI to predict trastuzumab resistance. Calibration was assessed using the Hosmer-Lemeshow test and graphically using a calibration plot. The observed frequency was calculated for each group. Predicted probability refers to the predicted probabilities generated by the model. Patients were grouped according to deciles of the predicted probabilities. Grouped patients refers to patients grouped at each decile of predicted probabilities generated by the model. A LOWESS regression was fit to all data points (blue line). (PDF 119 kb) [file 13058_2019_1160_MOESM4_ESM.pdf]
